# Secular trends in motor performance in Swiss children and adolescents from 1983 to 2018

**DOI:** 10.3389/fpubh.2023.1095586

**Published:** 2023-03-27

**Authors:** Elisa Knaier, Aziz Chaouch, Jon A. Caflisch, Valentin Rousson, Flavia M. Wehrle, Tanja H. Kakebeeke, Oskar G. Jenni

**Affiliations:** ^1^Child Development Center, University Children’s Hospital Zurich, Zurich, Switzerland; ^2^Division of Biostatistics, Center for Primary Care and Public Health (Unisanté), University of Lausanne, Lausanne, Switzerland; ^3^Children’s Research Center, University Children’s Hospital Zurich, Zurich, Switzerland; ^4^Department of Neonatology and Intensive Care, University Children’s Hospital Zurich, Zurich, Switzerland

**Keywords:** secular trends, secular changes, motor performance, children, adolescents, Zurich Neuromotor Assessment

## Abstract

**Introduction:**

Environmental changes, including globalization, urbanization, social and cultural changes in society, and exposure to modern digital technology undoubtedly have an impact on children’s activity and lifestyle behavior. In fact, marked reductions in children’s physical activity levels have been reported over the years and sedentary behavior has increased around the world. The question arises whether these environmental changes had an impact on general motor performance in children and adolescents. The study aimed to investigate secular trends of motor performance in Swiss children and adolescents, aged between 7 and 18 years, over a period of 35 years from 1983 to 2018.

**Methods:**

Longitudinal data on the five motor components of the Zurich Neuromotor Assessment (ZNA) – pure motor (PM), fine motor (FM), dynamic balance (DB), static balance (SB), and contralateral associated movements (CAM) – were pooled with cross-sectional data on PM and FM from eight ZNA studies between 1983 and 2018. Regression models were used to estimate the effect of the year of birth on motor performance and body mass index (BMI) measurements. Models were adjusted for age, sex, and socioeconomic status.

**Results:**

The secular trend estimates in standard deviation scores (SDS) per 10 years were − 0.06 [−0.33; 0.22, 95% Confidence Interval] for PM, −0.11 [−0.41; 0.20] for FM, −0.38 [−0.66; −0.09] for DB (−0.42 when controlled for BMI), −0.21 [−0.47; 0.06] for SB, and − 0.01 [−0.32; 0.31] for CAM. The mean change in BMI data was positive with 0.30 SDS [0.07; 0.53] over 10 years.

**Discussion:**

Despite substantial societal changes since the 1980s, motor performance has remained relatively stable across generations. No secular trend was found in FM, PM, SB, and CAM over a period of 35 years. A secular trend in DB was present independent of the secular trend in body mass index.

## 1. Introduction

Secular trends are changes in human performance or characteristics that occur in a population over decades or generations (1). They are explained largely by environmental factors (2–4). Best known are the secular trends in growth and intelligence from the 19th century on (2, 5–7). Since then, population measures of human growth such as height and weight have increased considerably in all developed countries (2, 8). These secular trends have also been associated with an accelerated maturation of individuals (2, 9). Furthermore, the populations’ intelligence quotient increased in the 20th century in both developed and developing countries, a change known as the Flynn effect (10). Such changes are thought to be caused by better nutrition, improved access to health care, more exposure to and quality of education, and other environmental factors (4). In recent years, however, secular trends in growth and intelligence seem to have slowed (11, 12), stabilized (11), or even reversed (4, 10). Again, environmental changes, including globalization, urbanization, social and cultural changes in society, and exposure to modern digital technology are believed to be the cause of these new trends.

These changes in environmental factors over decades undoubtedly have an impact on children’s activity and lifestyle behavior. In fact, marked reductions in children’s physical activity levels have been reported over the years (13); for instance, 46% less physical activity in girls and 23% less activity in boys were found over a 5-year period in British children aged 11–16 years in 2004 (14). Low activity levels might also explain the decline in the aerobic physical fitness of children and adolescents since the 1970s (15, 16). Furthermore, sedentary behavior has increased around the world (17) and is considered to be a serious public health problem in many countries (18–20). Factors such as school policies, student curricula, traffic safety rules, and the increase in digital technology use may have created an environment that promotes inactivity and sedentary behaviors, not just today but already by the end of the previous century (21–23). In fact, since the 1970s, television viewing has increasingly been replaced by the use of new electronic and screen-based entertainment media (24). The gaming era started in 1972 with the release of the first arcade machine, and subsequently, video games have evolved into a massive global industry that now play a central role in contemporary youth culture (25). For example, more than two thirds of children at age 5 years were reported by their mothers in the Millennium Cohort Study to play video games on weekdays (26). And since the first decade after the millennium, even more diverse digital technologies have been used for screen-based leisure activities (27), even at very early ages (28, 29).

All these environmental changes may have an impact on children’s health and skills. Indeed, studies indicate a positive association between the time that children spend playing video games and the incidence of obesity and an increased body mass index (BMI) (30, 31) as well as a negative relationship between electronic gaming time and participation in physical activities (32, 33). In contrast, video gaming is associated with enhancements in visuospatial and attention-related skills (34, 35). As early as 1983, Griffiths et al. found better hand–eye coordination in individuals with video-game experience than in subjects with no video-game experience (36). In fact, improved hand–eye coordination could have a positive impact on fine motor (FM) skills. Furthermore, faster reaction times in response to visual stimuli is reported in most video-game literature (37–39).

These findings raise the question whether the environmental changes in recent decades have also had an impact on general motor performance (MP). MP characterizes the individual performance of a wide range of basic motor skills but does not include motor fitness (40). Although children acquire basic motor skills naturally through maturational processes, a developmentally appropriate environment also promotes better MP through stimulation and practice (41). Furthermore, MP is influenced by several additional factors such as height, weight, BMI, waist circumference (42), sex (43, 44), ethnicity (45), socioeconomic status (SES) of the parents (46), and geographical location (44, 47).

Studies on secular trends in MP are limited and inconclusive. For example, a study by Roth et al. of various motor tasks in German preschoolers between 1973 and 2007 observed improvements such as in jumping performance, but balancing and throwing performance declined (48). Similarly inconclusive results were reported by Hardy et al. investigating Australian children between 9 and 15 years of age between 1997 and 2010 (49). In this study, running and catching performance improved slightly, but jumping performance declined. In contrast, Brian et al. found overall negative secular trends in MP in children from Belgium and the United States between 1997 and 2013 (50). The reasons for the inconsistent findings of previous studies may be the addition and/or exclusion of some motor tasks, modifications in the testing procedures over time, and the recruitment of children with differing characteristics such as SES. Thus, standardized test procedures are essential to detecting secular trends in human performance measures.

The goal of this study was to investigate secular trends in MP in Swiss children and adolescents over a period of 35 years from 1983 to 2018. Compared to previous studies, our data pool offered several novel aspects for identifying secular trends in MP. In particular, the data were gathered from a large study sample over 35 years with the same motor test instrument. Moreover, the motor test examined not only gross and fine motor skills but also motor abilities such as pure motor (PM) performance and movement quality. Secular trends in movement quality have never been examined before. Additionally, both BMI and SES were available to control for possible confounders. Increased sedentary screen time in combination with a decrease in physical activity since the 1980s led us to hypothesize negative secular trends in children’s gross motor skills. Conversely, due to the spread of digital media technology in recent decades we expected a positive secular trend in children’s FM skills.

## 2. Materials and methods

### 2.1. Data source

The Zurich Longitudinal Studies (ZLS) are a set of three Swiss longitudinal cohort studies (ZLS-1, ZLS-2 and ZLS-3) on children’s growth, health, and development [see (51)]. In the 1970s, the study investigators added the Zurich Neuromotor Assessment (ZNA) (52–54) to the study’s assessment procedure in order to investigate motor performance. Later, several additional studies were initiated (Table 1).

**Table 1:** Characteristics of studies and participants.

	Study	Reference	Test battery	*n* (7–18 years)	% females	Birth years	Test years	Type of study^†^
Step 1	ZLS-3	(51)	ZNA	264	49.7	1973–2001	1983–2018	Long
Step 2	ZLS-3	(51)	ZNA	264	49.7	1973–2001	1983–2018	Long
	ZLS-2	(51)	ZNA	95	47.7	1974–1979	1983–1997	Long
	ZNA norms	(52, 53)	ZNA	320	49.4	1981–1988	1995	Cross
	ZNA reliability	(74)	ZNA	55	54.5	1988–1993	1996–2001	Cross
	ZNA controls 1	(75, 76)	ZNA	35	34.3	1990–2000	2008–2009	Cross
	ZNA controls 2	(77)	ZNA	32	53.1	1996–2003	2013	Cross
	ZNA-2 norms	(55)	ZNA-2	365	49.3	1997–2009	2015–2017	Cross
	ZNA-2 controls	(Unpubl.)	ZNA-2	17	35.3	2002–2009	2017–2018	Cross

† Long, longitudinal; Cross, cross sectional.

Two steps were taken to evaluate secular trends in MP. In the first step, we included longitudinal data from the ZLS-3 (born between 1973 and 2001). Secular trends were investigated on five motor components of the ZNA. This choice of the ZLS-3 was motivated by several considerations. Firstly, the definition of motor tasks evolved over the years, and new norms were created for the ZNA (55). Thus, some motor components were no longer exactly comparable with those defined in the original ZNA. However, within the ZLS-3 study, the same norming system was used throughout all five components. Secondly, a consistent SES scoring system and complete BMI data were available in the ZLS-3, allowing secular trend estimates in MP and BMI to be adjusted for SES.

In the second step of the analysis, ZNA data were pooled from eight subsequent studies between 1983 and 2018 from children born between 1973 and 2009 (Table 1). The analyses focused exclusively on the PM and FM component as these are the only motor components that were assessed identically over time. Thus, data from the mid-1970s measured with the original ZNA could be compared with more recent data that were assessed with the revised and updated version, the ZNA-2 (55). This step substantially increased the sample size from 2000 onwards and extended the period in which secular trends may have occurred.

### 2.2. Participants

All participants were healthy and born full term. In total, 1,183 subjects from eight studies were included in this paper. They were born between 1973 and 2009 and tested at ages between 7 and 18 years in the period between 1983 and 2018; see Table 1 for details. Written informed consent was obtained from a primary parent or guardian and all participants 14 years and older. Participants younger than 14 years gave verbal consent. The studies were performed according to the Declaration of Helsinki and approved by the local ethics committee.

### 2.3. Instruments

#### 2.3.1. Zurich Neuromotor Assessment

The ZNA (54) was used to assess MP in children and adolescents. The ZNA is a standardized procedure to measure an entire range of MP in children and adolescents aged 5–18 years. In the ZNA, the speed of several motor tasks is timed, and quality of movements such as contralateral associated movements (CAMs) is assessed, subdivided into five motor components: FM, PM, dynamic balance (DB), static balance (SB), and CAMs (52, 53).

Recently, the original ZNA was revised and improved. The updated version is called ZNA-2, and new norms have been published (55). Both the ZNA and ZNA-2 are reliable test instruments and are described in more detail in Largo et al. (52, 53) and Kakebeeke et al. (55, 56), respectively.

#### 2.3.2. Socioeconomic status

SES data were collected through parental questionnaires with scores calculated by converting parental occupation and maternal education to a 6-point scale (51–53).

#### 2.3.3. Body mass index

Participants’ body weight and height were measured to the nearest 0.1 kg and 0.1 cm. Each participant’s weight in kilograms was divided by the square of their height in metres to calculate BMI (kg/m2).

### 2.4. Procedure

Each participant was tested individually in a quiet room. For more precise evaluation of MP (e.g., CAM), participants’ movements were recorded on digital camera. All study examiners had previously been trained in MP evaluation and were supervised by highly experienced examiners. Motor tasks were always administered in the same sequence: starting with FM, followed by PM and SB, and ending with DB.

### 2.5. Statistical analysis

In both steps, MP was converted into standard deviation scores (SDS) using norms from the original ZNA (52–54). An SDS is a continuous and standardized measure of MP, adjusted for age and sex, that is normally distributed in the population of typically developing children with a mean of 0 and an SD of 1. An SDS of 0 refers to average performance, and positive and negative values refer to above-average and below-average performances, respectively. BMI was converted into SDS using Swiss reference values (57).

The first step used ZLS-3 data to estimate secular changes with a linear mixed-effects model using longitudinal motor SDS as the dependent variable and age at the time of examination (categorized into three age groups: 7–8 years, 10–12 years, and 15–18 years), sex, SES, and the year of birth as predictors. Both SES and the year of birth were treated as continuous predictors. A linear trend was used to model the effect of the year of birth (i.e., secular trend). The SES effect was modelled with fractional polynomials of degree 1 (58) for PM and DB, whereas a linear fit was adequate for FM, SB, CAM, and BMI. An interaction term between the year of birth and age group was tested with a likelihood ratio test to detect any heterogeneous effect across age groups. This interaction term was never found to be statistically significant (*p* ≥ 0.095). Therefore, we report the estimates obtained from models in which the secular trend is assumed equal in all age groups. A random intercept was included in the model for each child to account for the longitudinal design. Additionally, an exponential correlation structure was added to capture a serial correlation between measurements taken on the same child at different ages. We computed 95% confidence intervals (CI) for the secular trend estimates for all five motor components and BMI. Additionally, Bonferroni-corrected CI that control for the family-wise error rate were also computed for the five motor components.

The second step included eight studies; two of these, ZLS-2 and ZLS-3, were longitudinal, whereas the others were cross-sectional. We chose to retain only one measurement per child to prevent results from being driven by these two longitudinal studies. We selected these single measurements using stratified random sampling with inclusion probabilities inversely proportional to the number of participants in each age group in the cross-sectional studies. This sampling strategy allowed measurements to be preferably selected from longitudinal studies within age groups that were less well covered in cross-sectional studies. The secular trend was then estimated with linear mixed-effects models using either PM or FM SDS as the dependent variable and incorporating study indicators as random intercepts. The age group, sex, and year of birth were entered as fixed effects. Note that SES could not be controlled for in this analysis because of the inconsistency with which SES were recorded in the various studies. Plots of predicted SDS as a function of the year of birth (for males aged 10–12 years) were used to visualize the magnitude of the secular trend with respect to the overall variability in the data. A parametric bootstrap procedure (59) with 2000 replications was performed to estimate 95% CI for the predicted average SDS. In both steps, a level of 5% was chosen to define statistical significance.

## 3. Results

**Figure 1:**
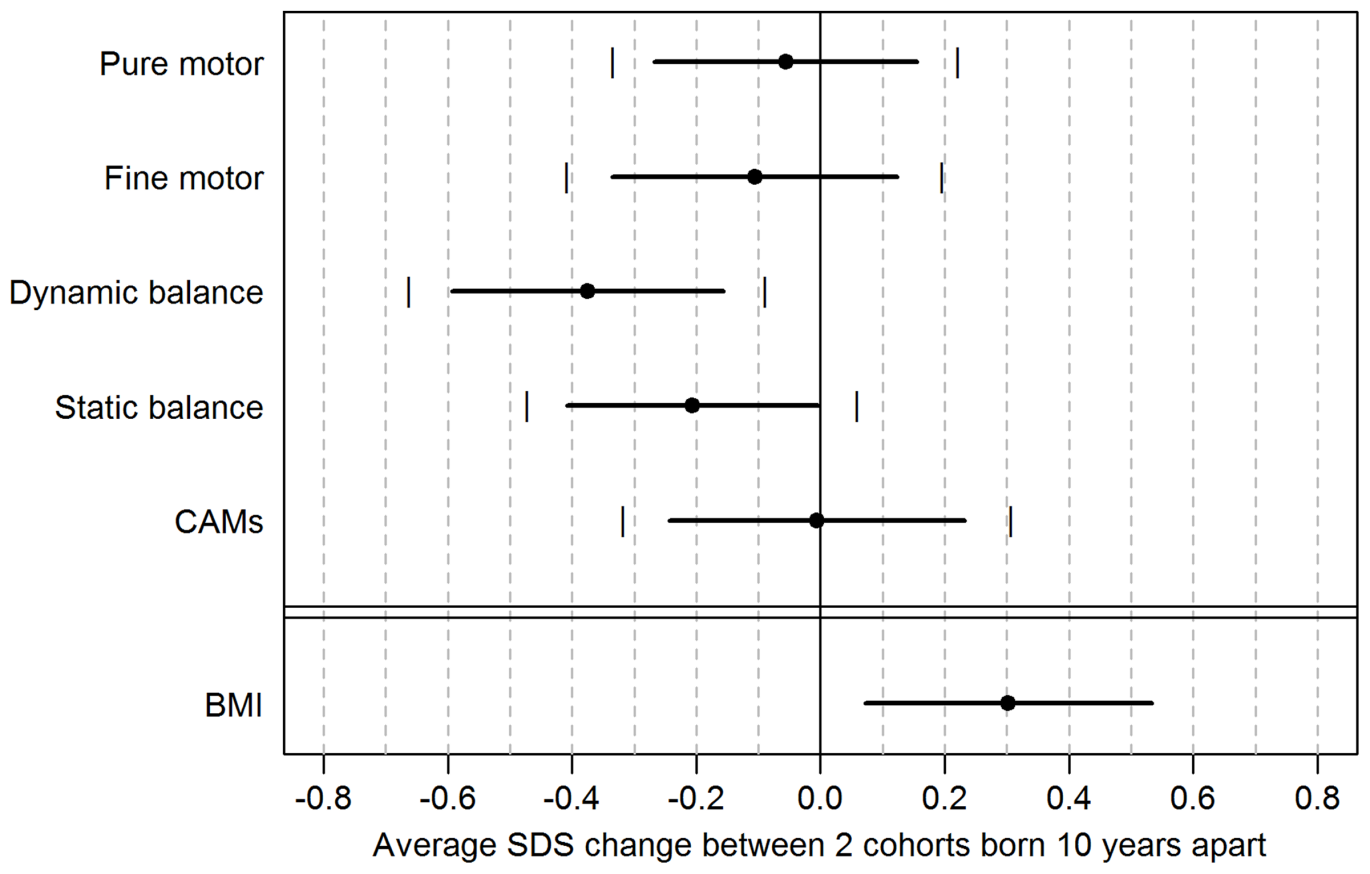
Estimated secular trend based on ZLS-3 data: average change in SDS between two cohorts born 10 years apart for five motor components of the ZNA and BMI, with 95% CI with (vertical lines) and without (horizontal lines) Bonferroni correction for multiple testing of motor components. All estimates are adjusted for age, sex, and SES. Positive values suggest that children born in year Y performed better and had higher BMI than peers of the same age and sex born in year Y-10. Negative values suggest that children born in year Y performed worse and had lower BMI than peers of the same age and sex born in year Y-10.

Table 2 and Figure 1 present the secular trend estimated from the first step of analysis, gathered from ZLS-3 data, for each motor component in the ZNA and for BMI. The secular trend is here expressed as an average change in SDS between two cohorts born 10 years apart. With the exception of DB, the CI for the estimated secular trends covered the value of zero in all motor components, whether accounting for multiple testing or not, indicating that our data are compatible with an absence of secular trend in four out of five motor components. The secular trend for DB was estimated as a difference of −0.38 without adjusting for BMI, 95%CI [−0.66; −0.09] between two cohorts born 10 years apart. When the trend estimate of DB was controlled for BMI SDS scores in addition to age, sex, and SES, the secular DB changes was − 0.42 SDS.

**Table 2:** Estimated secular trend based on ZLS-3 data: average change in SDS between two cohorts born 10 years apart for five motor components of the ZNA and BMI, with 95% CI with and without Bonferroni correction for multiple testing.

Dimension	Estimate	95% CI	Bonferroni corrected interval
**Motor components**			
Pure motor (PM)	−0.06	[−0.27; 0.16]	[−0.33; 0.22]
Fine motor (FM)	−0.11	[−0.34: 0.12]	[−0.41; 0.20]
Dynamic balance (DB)	−0.38	[−0.59; −0.16]	[−0.66; −0.09]
Static balance (SB)	−0.21	[−0.41; 0.00]	[−0.47; 0.06]
CAM	−0.01	[−0.24; 0.23]	[−0.32; 0.31]
**Anthropometric**			
BMI	0.30	[0.07; 0.53]	not applicable

All estimates are adjusted for age, sex, and SES. Positive values suggest that children born in year Y performed better and had higher BMI than peers of the same age and sex born in year Y-10. Negative values suggest that children born in year Y performed worse and had lower BMI than peers of the same age and sex born in year Y-10.

Furthermore, the secular trend estimated for BMI was positive (0.30 SDS) and statistically significant [0.07; 0.53], suggesting that over any period of 10 years, the BMI increased in the population.

**Figure 2:**
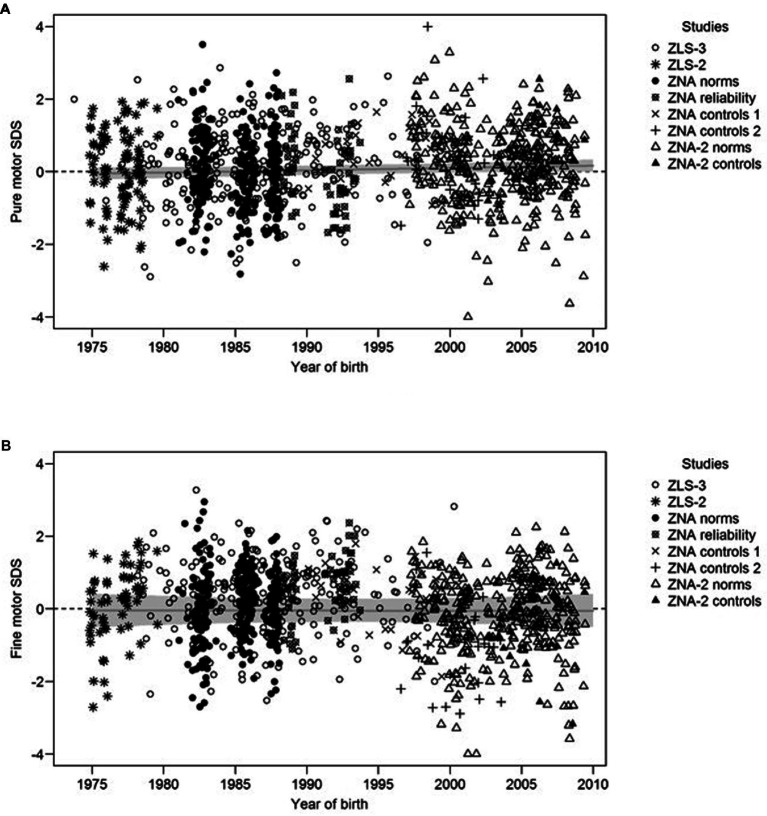
SDS of the PM **(A)** and FM **(B)** components as a function of the year of birth and the study. The regression lines refer to the predicted SDS for males in the mid age group (10–12 years) after controlling for systematic differences between studies using random intercepts. The secular trend corresponds to the slope of the regression lines. The grey area around regression lines represents a 95% CI estimated using 2000 parametric bootstraps.

Figure 2 illustrates the association between PM (A) and FM SDS (B) and the year of birth after merging data from all studies and selecting a single measurement per child for these studies with longitudinal data. The slope of the grey regression line refers to the estimated secular trend adjusted for the age group, sex, and random study effects. The secular trend estimates in SDS per 10 years are 0.06 [CI: 0.00; 0.12] and 0.00 [CI: −0.19; 0.19] for PM and FM, respectively. These estimates have narrower CI than those reported in Table 2 because they were obtained on a larger dataset. However, they still support the absence of secular trend from both PM and FM performance, which confirms results obtained with ZLS-3 data.

## 4. Discussion

This work provides estimates of secular trends in MP for Swiss children and adolescents, aged 7–18 years, born between 1973 and 2009 and tested over a period of 35 years (1983–2018). The main finding is that there is no evidence for the presence of any secular trend in PM, FM, SB, or CAM, neither when assessing longitudinal data (birth years 1973–2001) from the ZLS-3 cohort nor when assessing data from eight independent, cross-sectional studies in PM and FM over a broader period and with a larger sample (birth years 1973–2009). In contrast, we found a secular trend in DB decreasing by more than a third of a SDS every 10 years. Conversely, BMI data revealed a positive secular trend that is in line with previous studies investigating global trends (60) as well as changes in the Swiss population (57).

The first step of the study found the smallest average (statistically non-significant) change in SDS between two cohorts born 10 years apart in PM and CAM. This suggests that the average MP for these components has been remarkably stable over the years. This finding is supported by the second step of the analysis (shown in Figure 2A), which considered data merged from several studies with a broader time span. Figure 2A shows that the regression lines remain nearly horizontal, suggesting the absence of any secular trend from PM performance over a period of about 35 years. As far as we know, no comparable studies on secular trends include PM and movement quality tasks equivalent to CAM. Many motor batteries for children focus primarily on motor skills (61) that are strongly influenced by the environment and thus prone to changes and instability once the environment changes (62). In contrast, the ZNA also emphasizes measuring motor abilities, a concept that more directly reflects the child’s neurological development and is genetically influenced (56, 62, 63). Thus, these motor abilities remain more stable and less prone to environmental influences. This is especially true for the PM and CAM components. In PM, children perform isolated movements with their feet, hands, and fingers (53), movements that are usually not practiced during leisure time. CAMs reflect a child’s movement quality (52). The absence of secular trends from PM and CAMs is in line with the ZNA’s testing of fundamental aspects of neuromotor development, as one would suspect changes in neurological traits to take more than 35 years to occur.

Moreover, with only a minimal (statistically non-significant) average change over 10 years, the FM component also seems unaffected by a secular trend. This finding is supported by the second step of the analysis (shown in Figure 2B), which considered data merged from several studies spanning the decades after 2000. Figure 2B shows that the regression lines remain nearly horizontal, suggesting the absence of any secular trend in FM performance over about 35 years. This is contrary to our expectations, as we had hypothesized that new electronic and screen-based entertainment may promote overall better FM skills because these activities involve frequent movements in fingers and hands. Even though our cohort was most likely influenced by video games, computers, and consoles, neither a higher FM performance as hypothesized nor a decline as in Lin’s study (64) in children born after 2000 was detected in this study. Lin’s paper showed that access to a tablet actually lowers a child’s proficiency for FM precision, FM integration, and manual dexterity (64). Among young adults, however, time spent on the smartphone did not seem to influence measures of manual dexterity (65). In contrast, more than three decades earlier, Griffith’s study (36) showed enhanced hand-eye coordination in individuals with video-game experience. It is possible that the more manually operated video games had a different impact on general FM competence than today’s rather simple and intuitive touchscreens and therefore should not be considered comparable from the motor development perspective. Studies suggest that children born after 2008 show substantially more digital engagement than peers born a decade earlier (66). In fact, our study cohorts might be outdated regarding the most recent trends in digital engagement; the youngest children in our cohort were born in 2009. Future studies including children born after 2008 should bear the influence of digital engagement in mind and assess both FM skills and screen time when investigating secular trends.

The SB component shows a small (statistically non-significant) average change between cohorts born 10 years apart. In fact, the literature provides inconclusive results. Although balance tests performance showed a decline between 1992 and 2002 in Estonian children and adolescents (age 11–17), an increase was found in Lithuanian peers (67). A study with younger children (age 6–7) in Germany observed an relatively strong positive trend in balance performance (68). However, comparability between these studies is limited due to divergent methodological approaches.

In the current study, the largest and only statistically significant secular trend was found for the DB component. These findings are in line with results from other studies investigating vertical and horizontal jumping performances (16, 49, 69). Eberhardt et al.’s (16) review reported that performance in standing long jump increased constantly from the late 1950s until the 1980s and then stabilized before declining for another 15 years (16). Moreover, German preschool children performed significantly worse in a balancing backwards task in 2007 than did peers in 1985 (48). A possible explanation of the secular trend in DB could be the increasing BMI over generations, because a negative association of BMI with DB has been described in the literature (46, 70). Indeed, in parallel to the negative secular change in DB, we found a positive secular trend in BMI in the ZLS-3 data. However, when controlling the secular trend estimate of DB with BMI, the findings remained similar. Thus, BMI cannot directly explain the secular trend in DB in our cohort; consequently, other factors must be considered. As we suggested, increased sedentary screen-time behaviour (17) in combination with a decrease in physical activity (13) might play a role in explaining the decline in DB. In fact, Möller et al. found that longer television times, lower physical activity, and lower SES were also significantly associated with lower performance in strength, observed especially in the standing long jump task (46). It seems likely that children and adolescents’ modern lifestyles provide fewer opportunities to practice DB. Factors such as student curricula with less time for physical education classes and parental rules about safety, create an environment that promotes inactivity and sedentary behaviour (21–23). Lopes et al. (71) showed that increased sedentary behaviour predicts lower MP regardless of the child’s level of physical activity. This also reflects Biddle et al.’s (72) conclusion that sedentary and active behaviours can co-exist and do not necessary replace each other. For this reason, Hills et al. (22) postulated making stronger efforts to reduce the proportion of sedentary behaviour in childhood rather than primarily focusing on implementations that increase physical activity. Furthermore, sedentary behaviour is more likely to be associated with unhealthy dietary intake (73), which may raise the risk for obesity.

The analyses in this paper are not without limitations. It should be noted that we did not have many children with high BMI in this study, which may have underestimated the association between BMI and DB. Furthermore, neither data on physical activity nor media use was available in the ZLS-3, which would have given more information about possible confounders for changes in MP. To date, a major limitation of most studies is that there is no gold standard and high variability in the methods, tests, and procedures used to assess MP. Thus, standardized test procedures are essential to detecting secular trends in the future, along with consistent international reporting of information on SES, anthropometrics, physical activity, and screen-time.

We recommend that future research should monitor changes in MP (in particular in gross motor performance such as DB) and to potentially implement more recent data on MP in the future norm data as the recent ones might no longer be valid. It is important to continue to examine secular trends in the future, to identify declines in specific domains of MP, and to understand whether these may reflect changes in our society. Furthermore, it is essential to identify these declines as early and specifically as possible to mitigate or reverse any negative trend through appropriately tailored interventions. Because we found a secular decrease in DB as well as an increase in BMI, we suggest that health care professionals and educators more closely monitor gross motor performance and weight status of children and adolescents. Consequently, educational programs and interventions should be focused on gross motor skills in a variety of ways promoting children’s movement behavior.

In conclusion, the findings of this study indicate that FM, PM, SB and CAM – the majority of the components of MP – are unaffected by secular trends, whereas in contrast DB showed a secular trend independent of the secular trend in BMI.

## Data availability statement

The data that support the findings of this study are available from the corresponding author upon reasonable request.

## Ethics statement

The studies involving human participants were reviewed and approved by Cantonal Ethics Commission Zurich. Written informed consent to participate in this study was provided by the participant or participants’ legal guardian.

## Author contributions

EK conceived and designed the analysis of the data samples, contributed to data collection of some samples, wrote the manuscript. AC performed the statistical analysis of the entire data set. JC conceived and designed the analysis of the data sample, contributed to data collection to data collection of some samples, trained and supervised examiners. VR supervised the statistical analysis of the data. FW is the current project leader of the Zurich Longitudinal Studies (ZLS), contributed to data collection of some samples, reviewed and edited the manuscript. TK conceived and designed the analysis of the data sample, contributed to data collection of some samples, trained and supervised examiners, reviewed and edited the manuscript. OJ is the principal investigator of the ZLS and the Zurich Neuromotor Studies, reviewed and edited the manuscript. All authors contributed to the interpretation of the results, revised the manuscript critically, and approved the submitted version.

## Funding

This study was supported by the Swiss National Science Foundation, grant no 32003B_153273, and a fellowship grant from the Children’s Research Center of the University Children’s Hospital Zurich.

## Conflict of interest

The authors declare that the research was conducted in the absence of any commercial or financial relationships that could be construed as a potential conflict of interest.

TK, OJ, and JC are the publishers of the ZNA-2.

## Publisher’s note

All claims expressed in this article are solely those of the authors and do not necessarily represent those of their affiliated organizations, or those of the publisher, the editors and the reviewers. Any product that may be evaluated in this article, or claim that may be made by its manufacturer, is not guaranteed or endorsed by the publisher.

## Abbreviations

BMI: Body mass index
CAM: Contralateral associated movements
CI: Confidence interval
DB: Dynamic balance
FM: Fine motor
MP: Motor performance
PM: Pure motor
SB: Static balance
SDS: Standard Deviation Score
SES: Socioeconomic Status
ZLS: Zurich Longitudinal Studies
ZNA: Zurich Neuromotor Assessment
